# Biotic Interactions Shape the Ecological Distributions of *Staphylococcus* Species

**DOI:** 10.1128/mBio.01157-16

**Published:** 2016-10-18

**Authors:** Erik K. Kastman, Noelani Kamelamela, Josh W. Norville, Casey M. Cosetta, Rachel J. Dutton, Benjamin E. Wolfe

**Affiliations:** a Department of Biology, Tufts University, Medford, Massachusetts, USA; b Division of Biological Sciences, University of California, San Diego, La Jolla, California, USA

## Abstract

Many metagenomic sequencing studies have observed the presence of closely related bacterial species or genotypes in the same microbiome. Previous attempts to explain these patterns of microdiversity have focused on the abiotic environment, but few have considered how biotic interactions could drive patterns of microbiome diversity. We dissected the patterns, processes, and mechanisms shaping the ecological distributions of three closely related *Staphylococcus* species in cheese rind biofilms. Paradoxically, the most abundant species (*S. equorum*) is the slowest colonizer and weakest competitor based on growth and competition assays in the laboratory. Through *in vitro* community reconstructions, we determined that biotic interactions with neighboring fungi help resolve this paradox. Species-specific stimulation of the poor competitor by fungi of the genus *Scopulariopsis* allows *S. equorum* to dominate communities *in vitro* as it does *in situ*. Results of comparative genomic and transcriptomic experiments indicate that iron utilization pathways, including a homolog of the *S. aureus* staphyloferrin B siderophore operon pathway, are potential molecular mechanisms underlying *Staphylococcus*-*Scopulariopsis* interactions. Our integrated approach demonstrates that fungi can structure the ecological distributions of closely related bacterial species, and the data highlight the importance of bacterium-fungus interactions in attempts to design and manipulate microbiomes.

## IMPORTANCE

Decades of culture-based studies and more recent metagenomic studies have demonstrated that bacterial species in agriculture, medicine, industry, and nature are unevenly distributed across time and space. The ecological processes and molecular mechanisms that shape these distributions are not well understood because it is challenging to connect *in situ* patterns of diversity with mechanistic *in vitro* studies in the laboratory. Using tractable cheese rind biofilms and a focus on coagulase-negative *Staphylococcus* (CNS) species, we demonstrate that fungi can mediate the ecological distributions of closely related bacterial species. One of the *Staphylococcus* species studied, *S. saprophyticus*, is a common cause of urinary tract infections. By identifying processes that control the abundance of undesirable CNS species, cheese producers will have more precise control on the safety and quality of their products. More generally, *Staphylococcus* species frequently co-occur with fungi in mammalian microbiomes, and similar bacterium-fungus interactions may structure bacterial diversity in these systems.

## INTRODUCTION

From the early days of 16S rRNA clone libraries to the current groundswell of high-throughput metagenomics, one common pattern to emerge in studies of microbial community structure is the presence of numerous closely related species or strains of prokaryotes in the same habitat (1–3). These phylogenetic clusters have been observed in a wide range of environments, from marine microbiomes (4, 5) to our own human microbial landscapes (6). Many widespread bacterial species, including *Vibrio*, *Staphylococcus*, and *Streptococcus* species, commonly co-occur with closely related species or strains (6–8). Explaining these phylogenetic clusters has remained an important puzzle in microbial ecology because theory predicts that competitive exclusion should lead to dominance of only one major lineage when multiple phylogenetically similar lineages co-occur (9, 10). Identifying the ecological processes and molecular mechanisms that shape the distributions of closely related bacterial species can provide important tools to better manage and manipulate microbiomes in agriculture, medicine, and natural ecosystems.

Most previous attempts to explain the ecological distributions of bacterial species have focused on the role of abiotic niche partitioning and intraspecific or intrageneric biotic interactions (8, 11, 12), with much of our understanding coming from marine *Vibrio* species (5, 13–15) and oral biofilms (16, 17). For example, in coastal waters of the Atlantic Ocean, the coexistence of numerous genotypes of several species of *Vibrio* can be explained by temporal niche partitioning across seasons, preferences for particular microhabitats within seawater, and antagonistic interactions between ecologically defined populations (5, 18).

While niche specialization and intraspecific or intrageneric interactions may play important roles in driving coexistence in some microbiomes, biotic interactions between distantly related neighbors that span domains of life could also drive the composition within phylogenetic clusters. For example, in many microbiomes, bacteria coexist with diverse eukaryotic microbes, including fungi (19). Just as different plant species can mediate interactions between closely related insect herbivores that specialize on different resources (20), the distributions of coexisting bacterial species could be strongly regulated by the biotic niches created by co-occurring fungi. However, the causes and consequences of biotic interactions within microbiomes are poorly understood relative to impacts of the abiotic environment (3, 21). Examples of pairwise interactions between microbial species have been documented, including bacterium-bacterium interactions (22) and bacterium-fungus interactions (19). But it is unclear if these pairwise interactions dissected in the laboratory can help explain patterns of microbial diversity in multispecies microbial communities found in nature.

**FIG 1:**
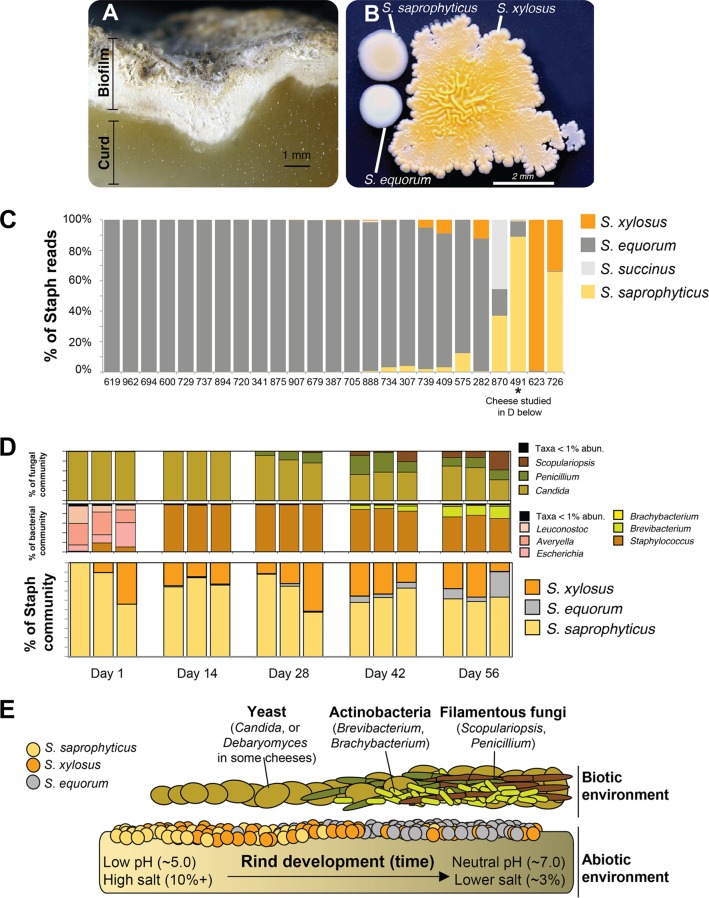
Ecological distributions of *Staphylococcus* species in cheese rind microbiomes. (A) A natural rind cheese showing the surface biofilm and curd. (B) Colonies of three of the most abundant CNS species found in cheese rinds were grown on plate count agar and photographed in natural light. Most strains of *S. saprophyticus* and *S. xylosus* are yellow or orange, while most strains of *S. equorum* are white. (C) Relative abundances of four CNS species in 25 cheese rind communities determined via metagenomic mapping of reads to reference genomes. Data are presented as relative abundances of reads that mapped to a *Staphylococcus* genome. Numbers at the bottom of the figure panel represent cheese sample identification numbers. (D) Relative abundances of four CNS species across a temporal sampling of 3 wheels of cheese aged over 56 days. Data for fungal and bacterial communities are from amplicon data presented in reference 26. Data for *Staphylococcus* communities were determined and are presented as described for panel C. (E) A model of *Staphylococcus* interactions with the biotic and abiotic environment during cheese rind development.

Cheese rind biofilms (Fig. 1A) provide a tractable system for exploring the role of biotic interactions in shaping distributions of closely related bacterial species. These simple communities are composed of various species of bacteria, yeasts, and molds that colonize the cheese surface during the aging process (23–25). Previous work has demonstrated that the accessibility, reproducibility, and tractability of these communities are ideal for dissecting the ecology of microbial communities (24). Here, we further demonstrate the utility of these communities by linking *in situ* observations of species abundance with *in vitro* experiments to determine what ecological processes shape the distribution of species within these microbial communities.

Coagulase-negative *Staphylococcus* (CNS) species are widespread but poorly understood members of cheese rind microbial communities. In a large-scale survey of 137 cheese rind communities, we found that *Staphylococcus* species were abundant (15% abundance on average across all cheese styles) and frequent (greater than 1% of the bacterial community in 67% of the cheeses surveyed) (24). Previously published studies performed in Europe and preliminary culture-based work performed in our laboratory have demonstrated that, across naturally aged cheeses and meats, four species of CNS are common: *S. equorum*, *S. xylosus*, *S. saprophyticus*, and *S. succinus* (26) (Fig. 1B). These species can also be commonly found co-occurring in animal microbiomes, including cow teats (27). Interestingly, these commonly co-occurring CNS species are very closely related members of the “*saprophyticus* cluster group” within the genus *Staphylococcus* (28–30), suggesting that they may have similar traits and share overlapping niches in the cheese rind microbiome. Ecological processes that shape the distribution of these commonly co-occurring species have not been identified.

By combining metagenomic data from *in situ* communities, *in vitro* reconstructions of experimental communities, and comparative genomics and transcriptomics, we address the following questions. (i) What are the ecological distributions of *Staphylococcus* species across cheese rind communities and during the development of cheese microbiomes? (ii) What ecological processes, including abiotic and biotic selection, determine the distributions of *Staphylococcus* species? (iii) Which potential molecular mechanisms underlie these ecological processes? The integrated ecological framework that we develop for dissecting the ecological distributions of these nonpathogenic *Staphylococcus* species could be applied to pathogenic *Staphylococcus* species in less-tractable microbiomes.

## RESULTS

### *Staphylococcus equorum* is the most abundant and widespread *Staphylococcus* species in cheese rind biofilms.

To measure the relative abundances of the four CNS species previously isolated from cheeses (*S. equorum*, *S. xylosus*, *S. saprophyticus*, and *S. succinus*), we used whole-genome shotgun metagenomic sequencing of rind microbiomes from 25 cheeses. Of these 25 metagenomes, 7 were previously published (24) and 18 were new metagenomes sequenced for this study (see Table S1 in the supplemental material). From each metagenome, we mapped 5.2 million 100-bp reads to an ~40-kb region of the reference genomes that provides careful discrimination between the four *Staphylococcus* species (see Materials and Methods). Percent relative abundance of each species was calculated as the number of reads mapped to a specific *Staphylococcus* species divided by the total number of reads mapped to all four *Staphylococcus* species genomes.

Across the 25 cheeses analyzed, *S. equorum* was the most abundant species (Fig. 1C), making up an average of 68.2% of the *Staphylococcus* reads detected across these rind communities. While 4 of 25 rind microbiomes were dominated by either *S. saprophyticus* or *S. xylosus*, *S. equorum* was dominant in 84% of the rind samples. *S. succinus* was very rare across the rinds sampled; it was detected in only 4 of the 25 rind communities. This metagenomic view of CNS in cheese supports previous culture-based surveys (26) suggesting that *S. equorum* is the most frequently encountered and locally abundant CNS species across cheeses. Given its widespread distribution, we hypothesized that certain traits in *S. equorum* promote its dominance in cheese and other fermented food microbiomes.

In addition to distribution across cheeses, we also investigated temporal patterns of abundance of the different *Staphylococcus* species during the development of a rind community. We chose a cheese (sample 491 in Fig. 1C) that had multiple species of *Staphylococcus* present so that we could identify any differences in colonization dynamics across the three species. Successional dynamics of all bacteria and fungi for this cheese have been previously described using amplicon metagenomic sequencing (24). Three replicate wheels of cheese were sampled at 1, 14, 28, 42, and 56 days after production, and the whole-genome shotgun metagenomic sequencing approach described above was used to determine relative abundances of *Staphylococcus* species over time. Unlike many of the previously described metagenomes, *S. equorum* did not dominate the *Staphylococcus* community at final stages of community development but showed a pattern of increasing relative abundance over time. At the early time points (day 1), reads of *S. equorum* were not detected whereas reads of *S. saprophyticus* and *S. xylosus* were detected. Over time, the relative abundance of *S. equorum* increased as the cheese aged, with the highest relative abundances detected at the final sampling time (day 56) (Fig. 1D). Note that these are relative abundance data, so it is impossible to distinguish between increasing abundance of *S. equorum* over time and decreasing abundance of the other species. But these data do suggest that *S. equorum* may be a late-colonizing species in cheese rind microbiomes.

### The most abundant species (*Staphylococcus equorum*) is a slow colonizer and a weak competitor.

To explain the distributions of *Staphylococcus* species, we began to test the role of major abiotic and biotic components that have been previously identified in cheese rinds, including interactions between *Staphylococcus* species (congeneric competition), interactions between *Staphylococcus* species and the abiotic environment (abiotic selection), and interactions between *Staphylococcus* species and other microbial species present in cheese rinds (biotic selection) (Fig. 1E).

One mechanism commonly used to explain coexistence in multispecies communities is the competition-colonization tradeoff (31–33). Theoretical models and experimental data have both shown that poor competitors are generally better at quickly colonizing an open niche before competitors arrive, while rapid colonizers are generally poor at competition with neighboring species. This competition-colonization tradeoff can lead to coexistence of the two types of species within a community (31–33). Based on the low rate of colonization by *S. equorum* observed in the temporal metagenomic survey, we predicted that *S. equorum* would be a poor colonizer of cheese. We also predicted that it would be a strong competitor to compensate for its poor colonization ability. To assess competition and colonization, we used *in vitro* cheese rind assays, where cheese curd agar (CCA) was used to mimic the cheese environment (24). We focused only on the three most frequently detected species (*S. equorum*, *S. xylosus*, *S. saprophyticus*) because *S. succinus* is rarely detected (<1% abundance across all cheeses sampled and across the time series).

**FIG 2:**
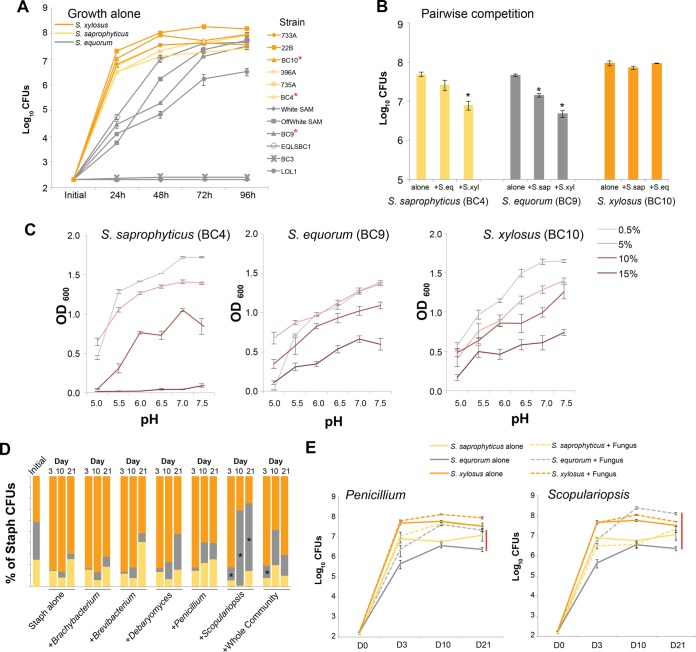
Ecological processes impacting the distributions of *Staphylococcus* (Staph) species in cheese rinds. (A) Colonization of different strains of *S. equorum*, *S. saprophyticus*, and *S. xylosus* on cheese curd agar. Points represent means (*n* = 3) ± 1 standard error. Strain (*F*_11,24_ = 115.19, *P* < 0.0001) and time of harvest (*F*_3,72_ = 151.09, *P* < 0.001) and their interaction (*F*_33,72_ = 16.187, *P* < 0.001) were significant based on a repeated-measures analysis of variance (ANOVA), with all *S. equorum* isolates having significantly lower growth than the *S. saprophyticus* and *S. xylosus* isolates based on Tukey’s honestly significant difference (HSD) test. Red asterisks next to strain names indicate the strains of each species used for the remaining experiments. (B) Pairwise competition of the three species of *Staphylococcus* (*S. equorum* [S.eq] strain BC9, *S. saprophyticus* [S.sap] strain BC4, and *S. xylosus* [S.xyl] strain BC10), with colors as defined for panel A. Bars represent means (*n* = 3) ± 1 standard error. Asterisks indicate significant differences in growth rates (*P* < 0.01) in the presence of an interspecific competitor compared to growth in the absence of an interspecific competitor (Dunnett’s test). (C) Growth of same species/strains as described for panel B over a range of pH and salt concentrations in BHI medium as measured by OD_600_ after 48 h. Points represent means (*n* = 3) ± 1 standard error. *S. xylosus* has a greater niche breadth (OD_600_ sum = 21.47 ± 0.50) than *S. saprophyticus* (OD_600_ sum = 19.187 ± 0.28) or *S. equorum* (OD_600_ sum = 19.08 ± 0.33) based on ANOVA with Tukey’s HSD test (*F*_2,5_ = 13.336, *P* < 0.01). (D) Relative abundances of the three species of *Staphylococcus* in experimental *in vitro* communities over 21 days. The three species were inoculated at initially identical densities and grown alone or with 5 single-species neighbors or an entire community containing all 5 neighboring species. Both biotic treatment (*F*_6,20_ = 48.268, *P* < 0.0001) and time of harvest (*F*_2,40_ = 29.310, *P* < 0.001) and their interaction (*F*_12,40_ = 12.528, *P* < 0.001) had significant effects on the abundance of *S. equorum*. Asterisks indicate significant stimulation of *S. equorum* compared to Staph-alone treatment based on Tukey’s HSD tests of absolute numbers of CFU. (E) Absolute abundances of the three species of *Staphylococcus* in the same experimental *in vitro* communities as described for panel D but only for molds *Penicillium* and *Scopulariopsis*. Solid lines represent the species growth in treatments without neighbors, while dashed lines represent the species growth in treatments with the fungal partner. Points represent means (*n* = 4) ± 1 standard error.

As expected based on the temporal metagenomic data, *S. equorum* was a slow colonizer of cheese compared to *S. xylosus* and *S. saprophyticus* (Fig. 2A). Over a 4-day period, 2 of 6 strains of *S. equorum* did not grow, while the remaining 4 strains grew much more slowly than *S. saprophyticus* and *S. xylosus*, with significant differences in growth between *S. equorum* and both *S. saprophyticus* and *S. xylosus* at 24 h of growth. These data corroborate the *in situ* late-colonization pattern observed in the temporal metagenomic study (Fig. 1D).

While the growth of *S. equorum* alone suggested that it is a slow colonizer of the cheese environment and may be at a disadvantage compared to the other two species, synergistic effects when grown with neighbors may allow it to outcompete the other two *Staphylococcus* species. Surprisingly, when grown in pairwise competition assays with the other two species of *Staphylococcus*, *S. equorum* was also a poor competitor. *S. xylosus* growth was not inhibited when grown with neighbors, but growth of both *S. saprophyticus* and *S. equorum* was significantly inhibited in the presence of neighboring species (Fig. 2B). *S. xylosus* had the greatest negative impact on neighboring species, suggesting that it is the strongest competitor in these communities. These *in vitro* growth and competition experiments suggest that *S. equorum*, the most widespread CNS species in cheese rinds, is the slowest colonizer and the weakest competitor of the three CNS species studied.

In some systems, specialization to different abiotic niches can allow species to coexist, and widespread species tend to have the largest abiotic niche breadth (34). We predicted that *S. equorum* might have a broader environmental niche breadth than the other two CNS species, and in environments that limit the growth of *S. xylosus* and *S. saprophyticus*, *S. equorum* can thrive. To test this prediction, we measured the growth of the three CNS species across gradients of salt and pH that span those found during the aging of cheeses (Fig. 2C; see also Fig. S1 in the supplemental material). Salt and pH are two of the most important drivers of bacterial growth in cheese microbiomes, and both of these environmental variables change during the aging of cheese as *S. equorum* becomes more abundant (24, 35). In contrast to our prediction, *S. xylosus* had the broadest niche breadth as determined by total growth across the range of pH and salt concentrations, with *S. equorum* and *S. saprophyticus* having more restricted niches (Fig. 2C; see also Fig. S1). Although the salt tolerance of *S. equorum* is greater than that of *S. saprophyticus* and comparable to that of *S. xylosus*, *S. saprophyticus* can grow at lower pH than both of the other species, which may explain why it dominates in the early stages of cheese rind development. These data suggest that, while these different *Staphylococcus* species do have different environmental tolerances, *S. equorum* does not have the broadest niche breadth and does not specialize in a particular abiotic niche. Therefore, abiotic niche breadth does not explain the widespread distribution of *S. equorum* in cheese rinds.

### Biotic interactions explain discordant *in situ* and *in vitro* patterns of abundance.

The contrasting *in situ* observations and *in vitro* experiments present an interesting ecological conundrum: how can a slow colonizer and weak competitor become the most abundant species in most cheese rind communities? We hypothesized that neighboring bacterial or fungal species could mediate competition between the *Staphylococcus* species through specific promotion of *S. equorum* or specific inhibition of *S. xylosus* and *S. saprophyticus.*

To test how biotic interactions impact the abundance of the three species, we repeated the same three-way competition experiment described above but added common bacterial (*Brachybacterium* and *Brevibacterium*) and fungal (*Scopulariopsis*, *Penicillium*, and *Debaryomyces*) species from cheese rinds to the 3-species *Staphylococcus* community. Addition of bacterial neighbors did not shift the abundance of *S. equorum* in the community (Fig. 2D). However, addition of fungi dramatically shifted the composition of the *Staphylococcus* community. Most strikingly, the mold *Scopulariopsis* shifted the community composition such that *S. equorum* became the dominant *Staphylococcus* species at the final time point (day 21), as was observed *in situ* (Fig. 2C). These shifts in relative abundance could be due to species-specific growth promotion of *S. equorum* or inhibition of the strong competitors *S. xylosus* and *S. saprophyticus*. Absolute data show the former; *Scopulariopsis* strongly stimulates growth of *S. equorum* but not growth of the other *Staphylococcus* species (Fig. 2E) (*S. equorum* abundance [*F*_1,20_] = 49.51, *P* < 0.001; *S. saprophyticus F*_1,20_ = 0.76, *P* = 0.39; *S. xylosus F*_1,20_ = 4.77, *P* = 0.04).

On the basis of our *in vitro* studies showing that *Scopulariopsis* strongly promotes *S. equorum* growth, we predicted that the presence of *S. equorum* and that of *Scopulariopsis* would be strongly positively correlated in naturally forming rind microbiomes. *In situ* data collected across the development of a cheese rind community (Fig. 1D) do show strong positive associations between *S. equorum* and the fungi *Scopulariopsis* (Spearman’s rho = 0.77, *P* = 0.006) and *Penicillium* (Spearman’s rho = 0.78, *P* = 0.005) (see Fig. S2 in the supplemental material). The presence of species of the *Actinobacteria* genera *Brevibacterium* (Spearman’s rho = 0.82, *P* < 0.001) and *Brachybacterium* (Spearman’s rho = 0.85, *P* < 0.001) is also strongly positively correlated with *S. equorum* abundance, even though these bacteria did not promote the growth of *S. equorum* experimentally (Fig. 2D), suggesting that *in situ* correlations of abundance do not always correctly predict experimental outcomes. Given that these data come from repeated sampling of the same three wheels of cheese, albeit from different locations across the rind surface (see Materials and Methods), these strong correlations should be interpreted cautiously due to lack of complete independence. But the data do provide a preliminary indication of the correspondence between the patterns of abundance in experimental interactions observed *in vitro* and *in situ*.

### Comparative genomics and transcriptomics point to potential molecular mechanisms underlying distributions of *Staphylococcus* species.

A specific interaction between the mold *Scopulariopsis* and *S. equorum* shifts the composition of the *Staphylococcus* community from dominance by a strong competitor, *S. xylosus*, to dominance by a weak competitor, *S. equorum*. To determine the potential molecular mechanism(s) underlying the bacterium-fungus interaction, we used comparative genomics of each of the *Staphylococcus* species alone as well as transcriptomics (RNA-sequencing or RNA-seq) of cocultures. Comparative genomics can pinpoint gene sets that are unique to species/strains that might explain ecological traits specific to a species (36, 37), while RNA-seq has the power to reveal the subset of genes and pathways that may underlie the outcomes of species interactions (38, 39).

**FIG 3:**
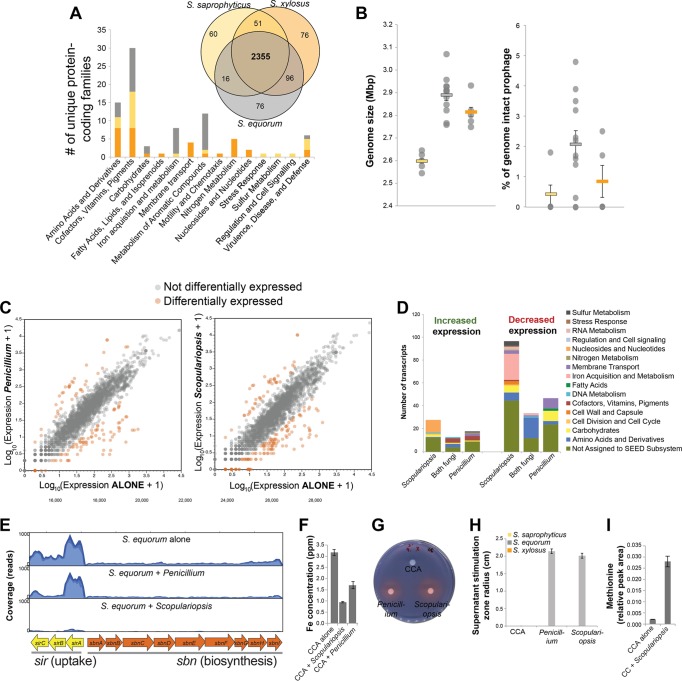
Putative molecular mechanisms underlying interactions between *Staphylococcus equorum* and fungi. (A) Comparative genomics of *Staphylococcus* species reveals species-specific genes, which are unevenly distributed across functional categories. Data are from 12 *S. equorum* (gray), 5 *S. saprophyticus* (yellow), and 5 *S. xylosus* (orange) strains. The Venn diagram shows overlap of clusters of protein-coding genes across the three species. The bar graph shows the distribution of species-specific genes (found in all strains of one species but not other species) across SEED subsystems. (B) Genome size estimates based on genome assemblies and percentages of prophage with intact genomes, as determined through PHAST annotations. (C) Changes in genome expression of *S. equorum* grown with the fungus *Penicillium* (left panel) or *Scopulariopsis* (right panel). Panels show scatter plots of global gene expression for *S. equorum* alone (*x* axis) versus with a fungus (*y* axis). Each dot represents a transcript from across the genome. Orange dots represent those transcripts that were differentially expressed when grown with the fungus (>5-fold change in expression; Rockhopper *q* value < 0.05). (D) Distribution of differentially expressed genes represented in panel C across SEED subsystems. (E) Representative read mapping at the staphyloferrin B operon from treatments with *S. equorum* alone (top), *S. equorum* plus *Penicillium* (middle), and *S. equorum* plus *Scopulariopsis* (bottom). Blue-shaded regions represent average coverage from read mappings. (F) Mean (± standard error) free iron concentration in cheese curd agar (CCA), CCA plus *Scopulariopsis*, and CCA plus *Penicillium*. All three conditions had significantly different iron concentrations based on ANOVA with Tukey’s HSD test (*F*_2,10_ = 10.30, *P* < 0.01). (G) Lyophilized and concentrated supernatants of CCA incubated without a fungus, with *Penicillium*, and with *Scopulariopsis* were spotted onto cellulose disks on chrome azurol S (CAS) medium. Shown is a representative plate after 1 day of incubation. (H) The supernatants used in the experiment whose results are shown in panel G were spotted onto cheese curd agar plates seeded with the three *Staphylococcus* species. The radius of zones of stimulation was measured around the cellulose disk after 3 days. (I) Mean methionine concentrations (± standard error) determined in cheese curd alone and cheese curd plus *Scopulariopsis* using capillary electrophoresis-time of flight mass spectrometry (CE-TOF MS). See Table S5 in the supplemental material for more details on metabolomics.

To determine gene sets that are unique to *S. equorum*, we compared protein-coding genes of 15 new genomes sequenced for this study that span the three *Staphylococcus* species, as well as 7 existing genomes (see Table S2 in the supplemental material). We found 76 protein-coding genes that were both present in all *S. equorum* strains and absent in *S. saprophyticus* and *S. xylosus* genomes (Fig. 3A; see also Table S3). One striking pattern to emerge is the enrichment of genes in *S. equorum* associated with iron acquisition and metabolism (Fig. 3A). Iron is one of the most limiting nutrients in cheese rinds (40), and *Staphylococcus* species have evolved various mechanisms for coping with low iron availability, including the production of siderophores (41). Much of the iron enrichment signal in *S. equorum* is due to the presence of a siderophore production and uptake operon that is homologous to the staphyloferrin B operon in *Staphylococcus aureus*. Staphyloferrin B is an alpha-hydroxycarboxylate siderophore that has been shown to be important in the growth and virulence of *S. aureus* (41). This operon is uncommon in the genus *Staphylococcus*; the entire operon is found only in *S. aureus*, *S. pseudintermedius*, and now *S. equorum*.

We also identified genomic features in *S. equorum* that might help explain why it is competitively inferior to *S. saprophyticus* and *S. xylosus*. The composition of genes that are absent in *S. equorum*, but present in *S. saprophyticus* and *S. xylosus*, does not suggest any obvious nutrition stress response or other deficiencies (see Table S3 in the supplemental material). But we did observe substantial variation in the distributions of prophage-encoding genes across the three species. The abundance of prophages within a genome has been shown to decrease the growth of some bacterial species, leading to the notion of a prophage burden (42–44). While *S. equorum* does have a significantly greater total genome size (*F*_2,19_ = 26.79, *P* < 0.001) and a higher total number of coding sequences (*F*_2,19_ = 15.70, *P* < 0.001), it also has a higher potential prophage burden, with a significantly higher percentage of total coding sequences being intact phage in *S. equorum* compared to *S. xylosus* and *S. saprophyticus* (Fig. 3B) (*F*_2,19_ = 4.04, *P* = 0.034). The high abundance of these prophages in *S. equorum* cannot not be explained by the lack of restriction-modification (R-M) systems that might help prevent phage infection; of the three species, *S. equorum* actually has the highest number of genes predicted to be components of R-M systems (see Table S2). None of these three CNS species are predicted to have functional clustered regularly interspaced short palindromic repeat (CRISPR) systems. It is unknown if and how these prophage can be activated to lyse cells, but the burden with respect to copying phage DNA during chromosome replication and the potential for phage-mediated lysis of cells may be greater for *S. equorum* than for *S. saprophyticus* and *S. xylosus*.

To better understand specific genes and pathways within *S. equorum* that respond to the strong stimulation by the mold *Scopulariopsis*, we used RNA-seq to identify genes that were up- and downregulated in the genome of *S. equorum* in the presence of fungi compared to the levels seen with growth of *S. equorum* alone. We compared the effect of *Scopulariopsis* (strongly promoted growth) on the *S. equorum* transcriptome to the effect of *Penicillium* (weakly promoted growth) to examine differences in the transcriptional responses to these two fungi. Complementing our comparative genomics findings, the RNA-seq data also point to a key role of iron acquisition in the biology of *S. equorum* on cheese, in particular when grown with *Scopulariopsis*. While the two fungal species had similar impacts on the transcriptional profiles of *S. equorum* in terms of the distributions of genes that were up- and downregulated (Fig. 3C; see also Table S4 in the supplemental material), *Scopulariopsis* had a unique impact on genes involved with iron acquisition and metabolism. A total of 23 genes associated with iron acquisition were downregulated when *S. equorum* was grown with *Scopulariopsis*, while no genes associated with iron acquisition were downregulated when *S. equorum* was grown with *Penicillium* (Fig. 3D; see also Table S4). No genes associated with iron acquisition were upregulated in either fungal treatment. The staphyloferrin B operon identified with comparative genomics was a major part of the strong iron response in our RNA-seq data set. Mean expression across the staphyloferrin B operon was significantly lower for both the biosynthesis components (*sbnABCDEFGHI*; *t* = 5.22, *P* < 0.001) and the uptake components (*sirABC*; *t* = 3.82, *P* < 0.01) of the operon when *S. equorum* was grown with *Scopulariopsis* (Fig. 3E).

To determine if and how fungi might alter iron availability in cheese, we measured free iron availability in our cheese curd medium. Both fungal species caused a decrease in free iron availability, but *Scopulariopsis* decreased iron availability to a lesser extent (Fig. 3F), potentially explaining the differences in iron-associated gene expression patterns in the RNA-seq datasets. This assay does not measure chelated forms of iron, such as iron in siderophore complexes. Siderophores produced by fungi may sequester free iron and may be used in place of endogenously produced staphyloferrin B. Supernatants of *Scopulariopsis* and *Penicillium* contained siderophores (Fig. 3G), as detected with the chrome azurol S (CAS) assay (45), and these supernatants stimulated the growth of *S. equorum*, but not *S. saprophyticus* or *S. xylosus*, on cheese curd agar (Fig. 3H), highlighting the specificity of these bacterium-fungus interactions. The fact that *Penicillium* supernatants also strongly stimulate *S. equorum* is surprising given that we did not see strong stimulation of *S. equorum* when *Penicillium* was grown with the three-species *Staphylococcus* community (Fig. 2E). This may be because actively growing *Penicillium* fungi have other impacts on neighboring species not captured by these supernatant assays.

Two other strong transcriptional responses were observed in the presence of both fungal species: expression of genes involved with methionine biosynthesis pathways strongly decreased, and expression of genes involved with thiamine biosynthesis increased (see Fig. S3 in the supplemental material). Filamentous fungi produce large amounts of extracellular proteases, and these proteases may release free amino acids that could stimulate the growth of neighboring bacterial species (35, 46). Using a global metabolomics screen, we observed a substantial increase in the levels of many free amino acids when *Scopulariopsis* was grown on cheese curd agar, including a greater than 10-fold increase in levels of methionine (see Table S5). The reason for an increase in expression of thiamine biosynthesis genes in *S. equorum* is less clear, but previous studies of bacterium-fungus interactions have observed that bacterially produced thiamine can support the growth of fungi that are thiamine auxotrophs (47).

## DISCUSSION

Biotic interactions between microbial species have been frequently proposed as potential drivers of microbiome diversity (21, 48–50). Putative biotic interactions have been inferred from observational metagenomic studies, where positive or negative associations between microbial taxa are used as a proxy for positive or negative interactions (49–51). This approach can be confounded by the structure of relative abundance data of most metagenomic studies and by shared niche preferences of noninteracting organisms (52). Microbial interactions within microbiomes have rarely been experimentally validated due to the challenges of creating ecologically relevant conditions in a laboratory environment and the limited ability to culture all members in complex multispecies microbiomes. When potential pairwise interactions have been carefully measured and dissected mechanistically (19, 53–55), they have rarely been linked to potential patterns of biodiversity and ecological processes occurring in naturally forming microbiomes (56). By integrating observations from metagenomics with *in vitro* community reconstructions and comparative omics approaches, we have demonstrated that very specific biotic interactions between bacteria and fungi can shape the composition of widely distributed and closely related bacterial species.

After comparing *in situ* distribution patterns of commonly isolated *Staphylococcus* species obtained through metagenomic sequencing with *in vitro* competition, colonization, and niche breadth data, we were presented with a striking paradox. Across many independently assembled cheese microbiomes sampled in the United States and Europe, the most widely distributed and locally abundant *Staphylococcus* species was a slow colonizer and a poor competitor and did not have the largest niche breadth. By measuring the impacts of neighboring species from the cheese community on the distributions of these *Staphylococcus* species, we identified fungal facilitation as a mechanism to explain the distribution of the weakest species, *S. equorum*. One specific fungus, the widely distributed *Scopulariopsis*, had the strongest effect on the composition of *Staphylococcus* biofilms. Our findings demonstrate a clear example of an interdomain interaction shaping the distribution of bacterial species within a microbiome and suggest that bacterium-fungus interactions can determine the distributions of widely distributed microbes.

**FIG 4:**
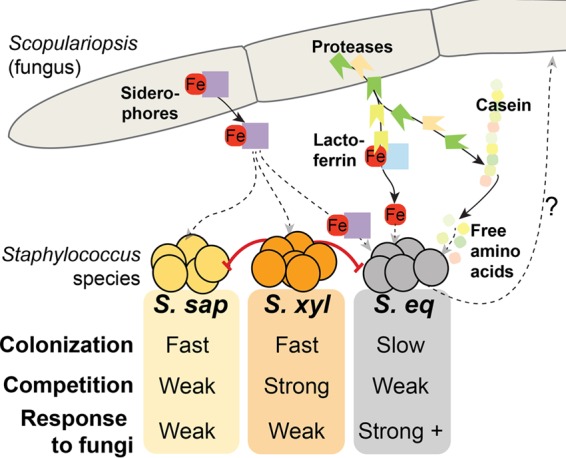
An overview of traits of cheese rind *Staphylococcus* species and potential mechanisms driving their interactions with fungi. Solid lines indicate interactions/mechanisms established in this work or in previous studies. Dashed lines indicate potential or unknown interactions/mechanisms.

Our comparative genomics and transcriptomics data suggest several putative mechanisms that shape the ecological distribution of *S. equorum* in cheese rinds. Comparative genomics did not reveal nutritional deficiencies that may explain lower growth rates of *S. equorum* in cheese, but the accumulation of prophages and large genome sizes of *S. equorum* may pose constraints on potential maximum growth rates. The fungi *Scopulariopsis* and *Penicillium* had different impacts on the *S. equorum* transcriptome, likely due to differences in metabolites produced by these two fungi or their impacts on the cheese environment (e.g., pH, nutrient availability). Both fungal species caused a decrease in expression of genes associated with amino acid acquisition and metabolism, while only *Scopulariopsis* caused a decrease in expression of iron acquisition genes. Much of the iron in cheese is bound by lactoferrin, an iron chelator present in milk (35), and microbes that colonize cheese produce siderophores to cope with iron limitation (40). Free amino acids are also in low supply in fresh cheese, as they are bound up in casein (35). *Scopulariopsis* may alter the availability of both iron and free amino acids by a variety of mechanisms (Fig. 4). Direct production of fungal siderophores by *Scopulariopsis* may relieve *S. equorum* of the costly production of the siderophore staphyloferrin B and potentially provide a unique iron source through cross-feeding. *Scopulariopsis* may also increase iron availability through release of chelated iron from lactoferrin through proteolysis, as has been suggested to occur with proteases of *Pseudomonas aeruginosa* (57–59). Proteolysis could also increase availability of free amino acids through casein degradation, potentially promoting the growth of slow-growing *S. equorum*. This current work highlights only a few potential molecular mechanisms, and the study did not fully test all potential mechanisms. More-detailed chemical analyses, including characterization of the fungal siderophores, are required to fully understand the molecular mechanisms driving the *Staphylococcus*-*Scopulariopsis* interaction.

Biotic interactions strongly altered the ecological distributions of the *Staphylococcus* species in these cheese biofilms, but other factors not manipulated in this study may also shape the abundance of *S. equorum* in microbiomes. High dispersal rates may counteract the slow colonization and poor competition traits of *S. equorum* and allow it to dominate cheese rind microbiomes. In the cheese production environment, high abundances of *S. equorum* in the raw milk used for cheesemaking or addition of *S. equorum* as a starter culture might allow high dispersal rates during the initial phase of cheese development. Previous surveys of microbial communities in raw milk have not indicated that *S. equorum* is particularly dominant relative to *S. saprophyticus* and *S. xylosus* (60–64), and *S. equorum* is not globally distributed as a starter culture in the cheese industry, suggesting that dispersal may not be important in explaining the widespread distribution of this species in cheese rinds. Future studies should examine the relative impacts of dispersal and biotic interactions in shaping the distribution of *S. equorum.*

While our work is driven by the search for basic principles of microbial community assembly, these results also have direct implications for the management of cheese microbiomes. One species of CNS studied, *S. saprophyticus*, causes urinary tract infections (UTIs) in healthy adults (65–69). While there is some confusion over whether the strains found in cheese and other foods can actually cause UTIs (65) and while there are no published reports of UTIs caused by eating cheeses, keeping *S. saprophyticus* out of cheese products should be a goal of cheese producers due to the potential for transfer of virulence factors or other mobile elements to the microbiome of cheese consumers. *S. equorum* is not considered a pathogen and has been suggested to be a potential starter culture given its previously described antilisterial activities (70, 71). Our work suggests that cheesemakers need to consider the management of co-occurring fungi in order to favor the growth of *S. equorum* over *S. saprophyticus*.

Fungi co-occur with bacteria in many microbiomes, including soils and the human body, but the ecology of bacteria and the ecology of fungi have traditionally been studied separately in the fields of bacteriology and mycology (19). Here we show that bacterium-fungus interactions can be important for explaining patterns of diversity within widespread microbial communities. Just as eukaryotic hosts (animals) can select for specific bacterial communities in host-associated microbiomes (72–74), fungi can select for specific bacterial communities in free-living microbiomes. While our work was performed using a synthetic community growing on an artificial substrate created by humans, fungal selection of bacterial diversity is likely to play a role in other microbiomes. For example, closely related *Staphylococcus* species co-occur in many human body sites, including the respiratory tract and skin microbiomes (6, 75–77). Various fungi can also co-occur in these same body habitats and may drive the distributions of pathogens such as *Staphylococcus aureus*. Future work exploring how fungi can shape the ecological distributions of bacterial species at fine phylogenetic scales can further elucidate the molecular mechanisms driving ecological patterns and processes in microbiomes.

## MATERIALS AND METHODS

### Isolation of strains.

Strains were isolated from rinds of cheeses or raw milk used for cheesemaking by serially diluting rind scrapings or milk samples on plate count agar with milk and salt (PCAMS) (24) with cycloheximide at a final concentration of 100 µg/ml. DNA was extracted from cells streaked on PCAMS using a PowerSoil DNA isolation kit (Mo Bio, Carlsbad, CA), and strains were initially identified to the species level using Sanger sequencing of the16S rRNA gene as previously described (24).

### Whole shotgun metagenomic library construction and mapping.

Whole shotgun metagenomic libraries from cheese rinds were constructed as described in reference 26 except that a New England Biolabs (Ipswich, MA) NEBNext Ultra DNA Library prep kit for Illumina was used according the manufacturer’s instructions. DNA was extracted from rinds using a PowerSoil DNA isolation kit, standardized to 1 µg in 50 µl of molecular-grade water, sheared to approximately 400 bp using a Covaris S220 focused ultrasonicator (Covaris, Woburn, MA), and used for library construction.

To determine the abundance of *Staphylococcus* species within each metagenome, 2.6 million 100-bp reads (for the survey across cheeses) or 1.7 million reads (for the survey over time from one cheese) were mapped to a conserved 39,648-kb region of the reference genome for each of the four CNS species (*S. equorum* strain BC9, *S. saprophyticus* strain BC4, *S. xylosus* strain BC10, and *S. succinus* strain BC15). This 39,648-kb region contains 44 protein-coding sequences and was chosen because preliminary cross-mapping studies for comparisons between the different *Staphylococcus* species demonstrated that this region could distinguish between these closely related species whereas the use of most other regions of the genome with highly conserved regions leads to false positives. Shotgun metagenomic data were mapped to reference genomes using the read mapper in Geneious 8.1.6. Reads were mapped with the following settings: minimum overlap identity = 97%; index word length = 14; map multiple best matches to none. Error rates based on mapping reads from individual genomes to all possible reference genomes were very low, ranging from 0.007% to 0.02%, demonstrating that this mapping approach provides precise discrimination of these four closely related species.

### *In vitro* growth, competition, and community assembly experiments.

Inocula for all *in vitro* experiments came from frozen glycerol stocks of brain heart infusion (BHI) broth liquid cultures that had been previously plated to determine CFU per microliter of inoculum. The main three strains used for experiments comparing *Staphylococcus* species were BC9 for *S. equorum*, BC4 for *S. saprophyticus*, and BC10 for *S. xylosus*. As our data in Fig. 2A demonstrate, each of these strains accurately represents the colonization and growth rates of each species. Depending on the *in vitro* experiment, different combinations of microbes were pooled in 1× phosphate-buffered saline (PBS) and inoculated onto the surface of 96-well microplates, with each well containing 150 µl of cheese curd agar (CCA) as previously described (24). For growth, competition, and community assembly experiments, inoculation for each species was approximately 200 CFU per well. For pairwise competition experiments, the total number of CFU of each species, even when grown alone, was 200 CFU. Growth-alone treatments had 200 CFU, while two-species treatments had 400 CFU in total. Postinoculation, microplates were sealed with a sterile, breathable film (Aeraseal; Excel Scientific, Victorville, CA). *In vitro* communities were incubated at 24°C for 3 days (to simulate cheese production facility conditions) and then at 14°C (to simulate cheese aging conditions) for the remainder of the incubation period (up to 21 days, depending on the experiment). To determine the CFU of each community member, communities were harvested by removing the entire CCA plug from a 96-well plate, homogenizing, and serial dilution plating as previously described (24).

### Niche screens.

The niche breadth of the three *Staphylococcus* species was determined by growing strains of each species in 200 µl of BHI broth under 24 different sets of pH and salt conditions that were distributed in grids across 96-well plates (see Fig. S1 in the supplemental material). Ten microliters of standardized inoculum at 20,000 CFU/µl of the appropriate strain was added to each well. Three replicate grids were assayed for each strain. After 48 h of growth with shaking at 200 rpm on a platform-shaking incubator at 24°C, the full volume of BHI broth in each well was pipetted up and down 5 times to remove any clumps, and optical density at 600 nm (OD_600_) was determined on a SpectraMax M5 plate reader.

### Genome sequencing and comparative genomics.

DNA was extracted using Mo Bio PowerSoil DNA extraction kits and streaks generated from a single bacterial colony grown for 2 to 3 days on PCAMS. Approximately 1 µg of purified genomic DNA (gDNA) was sheared using a Covaris S220 instrument to approximately 450 bp and was used as the input for a New England Biolabs (Ipswitch, MA) NEBNext Ultra DNA Library prep kit for Illumina. Libraries were spread across multiple sequencing lanes with other projects and were sequenced using 100-bp paired-end reads on an Illumina HiSeq 2500 instrument. Approximately 10 million reads were sequenced for each genome. Failed reads were removed from libraries, and reads were trimmed to remove low-quality bases and were assembled to create draft genomes using the *de novo* assembler in CLC Genomics workbench 8.0. Assembled genomes were annotated using RAST (78).

To compare the presence and absence of genes across strains and species, core and accessory genes were identified by assigning protein-coding sequences to functionally orthologous groups using the MultiParanoid method of the PanGenome Analysis Pipeline (PGAP) (79). Species-to-species orthologs were identified by pairwise strain comparison using BLAST with PGAP defaults: a minimum local coverage of 25% of the longer group and a global match of no less than 50% of the longer group, a minimum score value of 50, and a maximum *E* value of 1E^−8^. Multistrain orthologs were then found using MultiParanoid (80). Genes uniquely associated with each species were determined by filtering gene families to identify those present in all strains of a species and not present in any others, and functional annotations were determined using the RAST server and SEED subsystem annotations (78). Assessment of restriction-modification systems was conducted using SEED annotations, and assessment of potential CRISPR systems was conducted using CRISPRfinder (http://crispr.i2bc.paris-saclay.fr/).

### RNA-seq experimental setup, RNA isolation, library construction, and sequencing.

Experimental biofilms of *S. equorum* grown alone and with the two neighboring fungi were constructed by inoculating 20 ml of cheese curd agar in 100-mm-diameter petri dishes with 37,000 CFU of *S. equorum*. In the treatment using *S. equorum* alone, 100 µl of PBS was added to the *S. equorum* inoculum. In the fungal treatments, 100 µl of fungal inoculum was added to the *S. equorum* inoculum. *Scopulariopsis* was inoculated at ~3,700 CFU, while *Penicillium* was inoculated at ~370 CFU. The inoculation densities of the *S. equorum* and the fungi represented levels of bacterial and fungal densities similar to those that have been observed on cheese rinds (81, 82). We used different inoculation densities for the two fungi to take into account their very different growth rates; *Penicillium* reaches stationary growth in about 3 days whereas *Scopulariopsis* takes about 6 days to reach stationary growth. Previous preliminary experiments had demonstrated that using 1/10 the amount of the *Penicillium* inoculum produced similar densities of fungal growth across the *S. equorum* biofilms.

After 4 days of growth at 24°C, three replicate plates of each treatment (*S. equorum* alone, *S. equorum* plus *Penicillium*, and *S. equorum* plus *Scopulariopsis*) were harvested and used to create replicate RNA-seq libraries. Rind biofilms were harvested by scraping the cheese curd surface with a sterile razor blade to remove most of the microbial biomass. Harvested biofilms were stored in RNAProtect reagent (Qiagen) to stabilize mRNA and were frozen at −80°C. RNA was extracted using a standard phenol-chloroform protocol adopted from transcriptomics work in gut microbiomes (83). This protocol uses a standard bead-beating step in a lysis buffer to release cell contents from pelleted biofilms in RNAProtect. To ensure that the RNA was of high quality and not degraded, 500 ng of each RNA preparation was run and visualized on a 1.5% agarose gel. DNA was removed from the nucleic acid pool using a TURBO DNA-free kit (Life Technologies), and 5S/tRNA and rRNA were depleted using MEGAClear (Life Technologies) and RiboZero (Illumina) kits, respectively. To remove both yeast and bacterial rRNA, yeast and bacterial rRNA probes from the RiboZero kits were mixed 1:2 and used for rRNA depletion. To confirm that the samples were free of DNA contaminants, a PCR of the 16S rRNA was conducted with standard 16S primers (27f and 1492r).

RNA-seq libraries were constructed from purified mRNA using a NEBNext Ultra RNA Library prep kit for Illumina (New England Biolabs) and the manufacturer’s instructions and were sequenced using paired-end 100-bp reads on an Illumina HiSeq system in rapid run mode by the Harvard Bauer Core Facility. About 16 million reads were sequenced for each library. Only forward reads of the paired ends were used for analysis.

Analysis of RNA-seq libraries, including read mapping, normalization, and quantification of transcript abundances, was done using Rockhopper version 1.3.0 (84) with default settings. The *S. equorum* strain BC9 genome was concatenated and used as the reference genome for read mapping. Expression values were normalized using the upper quartile of gene expression. We considered differentially expressed genes to be those that exhibited a greater than 5-fold change in expression and that were deemed significantly different based on a *q* value of <0.05. Rockhopper’s *q* values are *P* values adjusted for the false-discovery rate using the Benjamini-Hochberg procedure.

### Fungal supernatant siderophore assay and growth stimulation assays.

To determine if fungi that promoted the growth of *S. equorum* produced siderophores and if supernatants of these fungi contained compounds that could stimulate bacterial growth, we grew the strains of *Penicillium* and *Scopulariopsis* described above in static liquid cheese curd (2% cheese curd, as opposed to 10% in solid medium) for 2 weeks at 24°C to obtain fungal supernatants. The cleared supernatant from this liquid was filter sterilized, frozen at −80°C, lyophilized, and then resuspended in sterile water. As a control, we also lyophilized 2% liquid cheese curd medium that had not been conditioned by any organism. For each of the three supernatant powders (control, *Penicillium*, *Scopulariopsis*), we added 2 mg of powder to 2 ml of sterile water for the experiments described below.

The chrome azurol S (CAS) assay was used to determine if fungal supernatants contained siderophores. We placed three sterile cellulose disks on the surface of a petri dish containing 20 ml of solid CAS assay medium (45). To the sterile cellulose disks, we added 20 µl of the hydrated cheese curd control powder that was not conditioned, the *Scopulariopsis* supernatant, or the *Penicillium* supernatant. After incubation in the dark at 24°C for 48 h was performed, we noted the presence of orange halos around the cellulose disks, indicating iron removal from the CAS assay blue dye complex.

We used the same approach to detect stimulation of *Staphylococcus* species by supernatants except that PCAMS or 10% cheese curd agar plates were used instead of the CAS assay medium. Standard (100-mm-diameter) petri dishes were seeded with approximately 75,000 CFU of one of the three *Staphylococcus* species to create a lawn of bacteria that could respond to the supernatant treatments. Three sterile cellulose disks were placed on the lawns, and 20 µl of control, *Scopulariopsis* supernatant, or *Penicillium* supernatant was added to one of the three disks. Three replicate plates for each species were used. Zones of stimulation were recorded after 3 days of growth, and the radius of stimulation zones was measured for the CCA plates after inverting photos of the plates to improve the ability to see colonies on the opaque cheese curd surface.

### Metabolomics and iron assays.

Cheese curd distributed in standard petri dishes was inoculated with either 100 µl of PBS or 100 µl of PBS containing ~3,700 CFU of *Scopulariopsis* (as in the RNA-seq experiments described above). After 10 days of growth, three replicate plates were harvested from each treatment and frozen until analysis by Human Metabolome Technologies (HMT) Inc. (Yamagata, Japan). Approximately 30 mg of each sample was mixed with 50% (vol/vol) acetonitrile in water, the mixture was homogenized, and the supernatant was filtered through a 5-kDa-cutoff filter (Ultrafree-MC-PLHCC; Human Metabolome Technologies Inc.) to remove macromolecules. The samples were then subjected to capillary electrophoresis-time of flight mass spectrometry (CE-TOF MS) for analysis of sugars, amino acids, nucleotides, and other ionic metabolites or to liquid chromatography-time of flight mass spectrometry (LT-TOF MS) for analysis of fatty acids, steroids, and other lipids using previously described analytical conditions and internal standards (85–87). Peaks were assigned to putative metabolites based on HMT’s standard library on the basis of *m*/*z* data and migration time (in CE-TOF MS) or retention time (in LT-TOF MS). Welch’s *t* test was used with false-discovery-rate correction of *P* values using the Benjamini-Hochberg procedure to identify metabolites that were significantly increased or decreased in abundance in the presence of *Scopulariopsis* on the basis of the relative peak area (metabolite peak area/ internal standard peak area × sample amount) for each metabolite.

To determine the concentration of available iron in the cheese curd medium, replicate plugs of cheese curd from the 96-well assays described above were removed from wells without microbes present (Control), where *Scopulariopsis* was inoculated (+*Scopulariopsis*), and where *Penicillium* was inoculated (+*Penicillium*) at the densities used in the community experiments described above. Iron concentrations in homogenates of these cheese curd samples were determined using an iron colorimetric assay kit (BioVision Inc.) according to the manufacturer’s instructions, except that 0.1 vol of 20% SDS was added to each sample to minimize interference from protein precipitation.

### Accession number(s).

Metagenomic libraries have been deposited in MG-RAST with accession numbers listed in Table S1 in the supplemental material. Assembled genome sequences have been deposited as whole-genome-sequencing (WGS) submissions to NCBI with accession numbers listed in Table S2. Raw RNA-seq reads and differential expression data have been submitted to the NCBI GEO database with GEO accession number GSE75505 (samples GSM1958050 through GSM1958058).

## SUPPLEMENTAL MATERIAL

Figure S1  Abiotic niche assays for more strains of each *Staphylococcus* species. Heat maps represent the growth of each strain across a gradient of salt and pH values as measured by OD_600_ after 48 h. Data for BC9, BC4, and BC10 are presented in more detail in Fig. 2. Data represent means of results of 3 replicates. Figure S1 relates to Fig. 2. Download Figure S1, TIF file, 0.9 MB

Figure S2  Spearman’s rank correlation coefficients of relative abundances of *Staphylococcus* species (from shotgun metagenomic data) and relative abundances of other members of the cheese rind community (from amplicon sequencing data). Correlations highlighted in bold are statistically significant (*P* < 0.01). See Results for discussion of caveats with respect to interpreting these data. Figure S2 relates to Fig. 1 and 2. Download Figure S2, TIF file, 0.9 MB

Figure S3  (A) The thiamine biosynthesis pathway. The part of the pathway highlighted with thick lines was differentially expressed (green = increased expression, red = decreased expression) in *S. equorum* grown with fungi. Small yellow boxes indicate if differential expression was observed with just *Penicillium* (P), with just *Scopulariopsis* (S), or with both *Penicillium* and *Scopulariopsis* (PS). (B) The methionine biosynthesis pathway. Notations are the same as those described for panel A. Figure S3 relates to Fig. 3. Download Figure S3, TIF file, 1.9 MB

Table S1  Shotgun metagenome metadata and features. Table S1, XLSX file, 0.1 MB

Table S2  Genome metadata and statistics. Table S2, XLSX file, 0.03 MB

Table S3  Gene clusters identified by pan-genome analysis pipeline (PGAP) present in all strains from each species. Table S3, XLSX file, 1 MB

Table S4  Significantly differentially expressed genes in plus-*Penicillium* and plus-*Scopulariopsis* treatments based on results of RNA-seq experiments. Table S4, XLSX file, 0.1 MB

Table S5  Metabolites detected in cheese curd agar (CCA) with and without *Scopulariopsis* by the use of capillary electrophoresis-time of flight mass spectrometry (CE-TOF MS) and liquid chromatography-time of flight mass spectrometry (LT-TOF MS). Table S5, XLSX file, 0.1 MB
