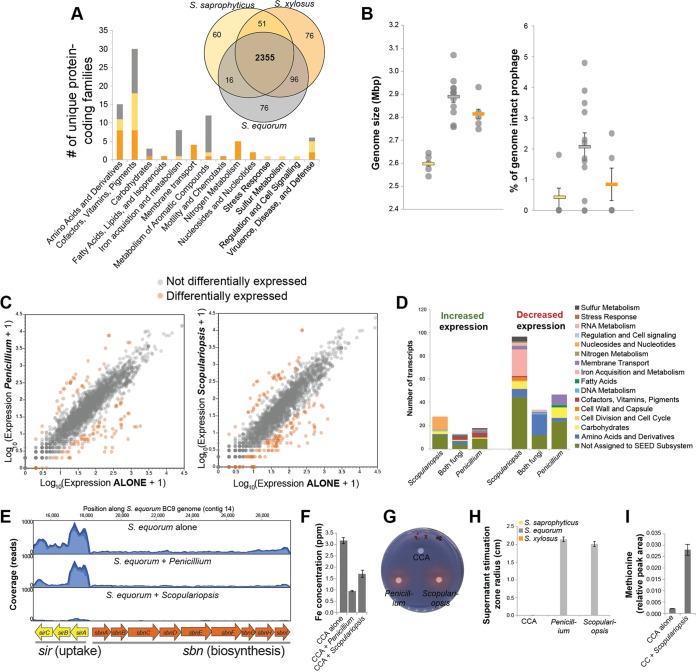# Erratum for Kastman et al., “Biotic Interactions Shape the Ecological Distributions of *Staphylococcus* Species”

**DOI:** 10.1128/mBio.00329-17

**Published:** 2017-03-21

**Authors:** Erik K. Kastman, Noelani Kamelamela, Josh W. Norville, Casey M. Cosetta, Rachel J. Dutton, Benjamin E. Wolfe

**Affiliations:** a Department of Biology, Tufts University, Medford, Massachusetts, USA; b Division of Biological Sciences, University of California, San Diego, La Jolla, California, USA

## ERRATUM

Volume 7, no 5, https://doi.org/10.1128/mBio.01157-16, 2016. In the Results section (PDF page 7), two errors were made in Fig. 3 that might cause confusion but do not change the major conclusions of the work. In Fig. 3E, labels that were intended to indicate the base pair position of genes in the staphyloferrin B operon along a contig in the *Staphylococcus equorum* BC9 genome were floating above the figure. In Fig. 3F, the *x* axis labels “CCA + *Penicillium*” and “CCA + *Scopulariopsis*” were switched. The revised Fig. 3 contains corrected versions of those labels.

**Figure:**