# Supplementary figures and images for: Biotic Interactions Shape the Ecological Distributions of Staphylococcus Species
Source: mBio. 2016 Oct 18;7(5):e01157-16. doi: 10.1128/mBio.01157-16 (PMC5082897; doi:10.1128/mBio.01157-16)

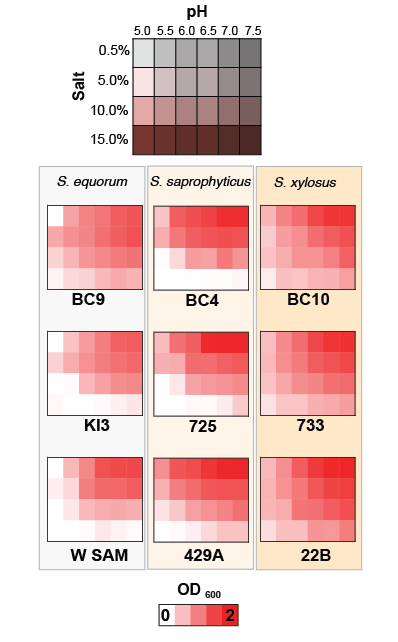

Supplement: Figure S1 — Abiotic niche assays for more strains of each Staphylococcus species. Heat maps represent the growth of each strain across a gradient of salt and pH values as measured by OD600 after 48 h. Data for BC9, BC4, and BC10 are presented in more detail in Fig. 2. Data represent means of results of 3 replicates. Figure S1 relates to Fig. 2. Download [file mbo005163030sf1.tif]

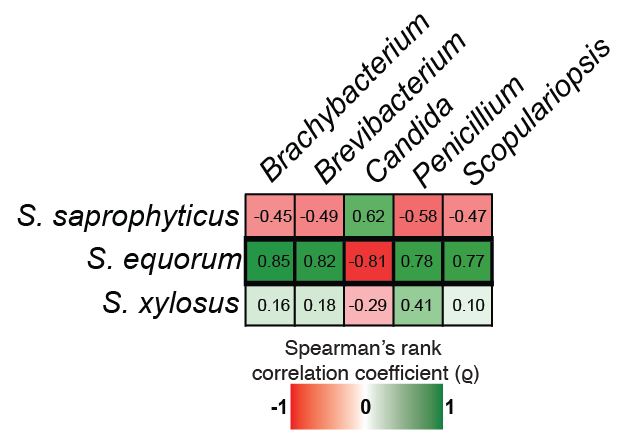

Supplement: Figure S2 — Spearman’s rank correlation coefficients of relative abundances of Staphylococcus species (from shotgun metagenomic data) and relative abundances of other members of the cheese rind community (from amplicon sequencing data). Correlations highlighted in bold are statistically significant (P < 0.01). See Results for discussion of caveats with respect to interpreting these data. Figure S2 relates to Fig. 1 and 2. Download [file mbo005163030sf2.tif]

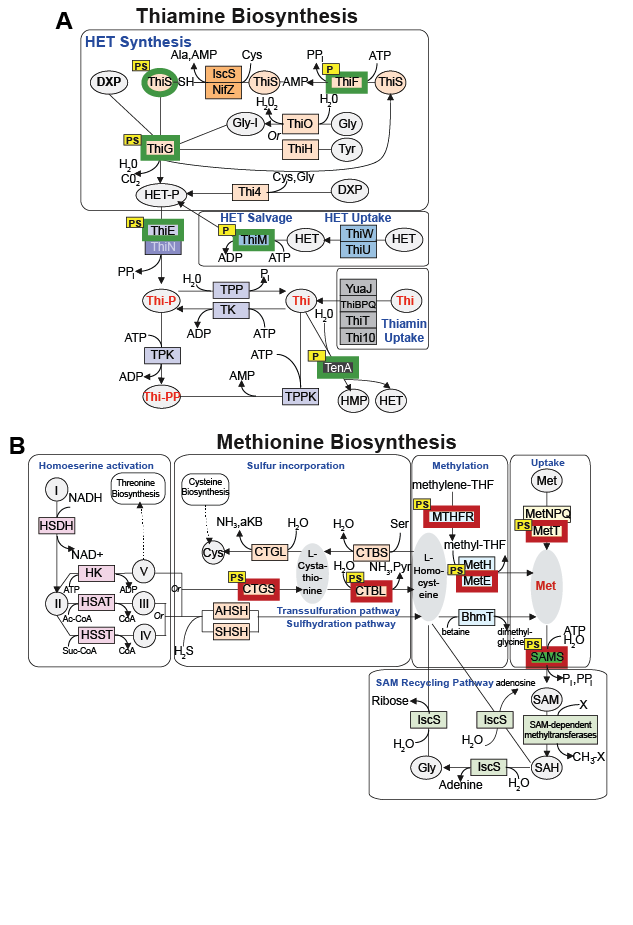

Supplement: Figure S3 — (A) The thiamine biosynthesis pathway. The part of the pathway highlighted with thick lines was differentially expressed (green = increased expression, red = decreased expression) in S. equorum grown with fungi. Small yellow boxes indicate if differential expression was observed with just Penicillium (P), with just Scopulariopsis (S), or with both Penicillium and Scopulariopsis (PS). (B) The methionine biosynthesis pathway. Notations are the same as those described for panel A. Figure S3 relates to Fig. 3. Download [file mbo005163030sf3.tif]
